# Mortality in the first six months among HIV-positive and HIV-negative patients empirically treated for tuberculosis

**DOI:** 10.1186/s12879-019-3775-z

**Published:** 2019-02-11

**Authors:** Helena Huerga, Gabriella Ferlazzo, Stephen Wanjala, Mathieu Bastard, Paolo Bevilacqua, Elisa Ardizzoni, Joseph Sitienei, Maryline Bonnet

**Affiliations:** 1 Epicentre, Paris, France; 2 Médecins Sans Frontières, Paris, France; 3 Médecins Sans Frontières, Nairobi, Kenya; 4 Institute of Tropical Medicine, Antwerp, Belgium; 5 Ministry of Health, Nairobi, Kenya; 6 IRD UMI 233 TransVIHMI - UM – INSERM U1175, Montpellier, France

**Keywords:** Tuberculosis, Diagnosis, Mortality, HIV, Resource-constrained, Empirical treatment

## Abstract

**Background:**

Empirical treatment of tuberculosis (TB) may be necessary in patients with negative or no Xpert MTB/RIF results. In a context with access to Xpert, we assessed mortality in the 6 months after the initial TB consultation among HIV-positive and HIV-negative patients who received empirical TB treatment or TB treatment based on bacteriological confirmation and we compared it with the mortality among those who did not receive TB treatment.

**Methods:**

This prospective cohort study included consecutively adult patients with signs and symptoms of TB attending an outpatient TB clinic in Western Kenya. At the first consultation, patients received a clinical exam and chest X-ray. Sputum was collected for microscopy, Xpert and *Mycobacterium tuberculosis* complex (MTB) culture. Patients not started on TB treatment were reassessed after 5 days. All patients bacteriologically confirmed (positive Xpert or culture) received TB treatment. Empirical treatment was defined as a decision to start TB treatment without bacteriological confirmation. Patients were reassessed after 6 months.

**Results:**

Of 606 patients included, 344/606 (56.8%) were women. Median age was 35 years [Interquartile Range (IQR):27–47] and 398/594 (67.0%) were HIV-positive. In total, 196/606 (32.3%) patients were Xpert- or culture-positive and 331/606 (54.6%) started TB treatment. Overall, 100/398 (25.1%) HIV-positive and 31/196 (15.8%) HIV-negative patients received empirical treatment. Mortality in the 6 months following the first consultation was 1.6 and 0.8/100 patient-months among HIV-positive and HIV-negative patients respectively. In the multivariate analyses, TB treatment - whether empirical or based on bacteriological confirmation- was not associated with increased mortality among HIV-positive patients (aHR:2.51, 95%CI:0.79–7.90 and aHR:1.25, 95%CI:0.37–4.21 respectively). However, HIV-negative patients who received empirical treatment had a higher risk of mortality (aHR:4.85, 95%CI:1.08–21.67) compared to those not started on treatment. HIV-negative patients treated for TB based on bacteriological confirmation did not have a different risk of mortality (aHR:0.77, 95%CI:0.08–7.41).

**Conclusions:**

Our findings suggest that in a context with access to Xpert, clinicians should continue using empirical TB treatment in HIV-positive patients with signs and symptoms of TB and negative Xpert results. However, differential diagnoses other than TB should be actively sought before initiating empirical TB treatment, particularly in HIV-negative patients.

## Background

Tuberculosis (TB) is one of the main killers in sub-Saharan Africa particularly in contexts with high HIV prevalence [1]. Incident TB has been strongly associated with increased mortality during antiretroviral treatment (ART) [2]. In addition, in post-mortem studies among HIV infected patients, TB remained undiagnosed at death in 46% of the TB cases [3].

GeneXpert MTB/RIF (Xpert) represents a major step forward in the diagnosis of TB [4]. Despite the increasing use of this assay in peripheral health facilities in the last several years [5, 6], and the development of Xpert MTB/RIF Ultra, a new improved version for MTB detection [7], TB diagnosis is still largely based on other diagnostic tools that have limited performance, such as clinical signs, sputum smear-microscopy, response to antibiotic trial, and chest X-ray [8–10]. In addition, the sensitivity of the Xpert is around 89%, decreasing to 79% in HIV-positive patients [11]. Therefore, two situations still require empirical TB treatment: patients with a negative Xpert result in a context where Xpert is available, and patients with no access to Xpert or delayed Xpert results [12, 13]. Although not demonstrated in clinical trials, Xpert has generated expectations regarding a possible decrease of mortality as patients could be more accurately and rapidly diagnosed and treated for TB [14–17]. On the other hand, it has also been hypothesized that empirical treatment could avoid deaths particularly in severely ill patients or those with higher risk of mortality such as HIV-positive patients. However, findings from different studies are not consistent [18, 19]. The question of whether empirically-treated patients will have a different risk of mortality compared to those who receive TB treatment guided by bacteriological result has not been fully solved in HIV-positive patients, and is poorly documented in HIV-negative patients [20–22].

In 2012, the Xpert MTB/RIF assay was introduced in Homa Bay District Hospital, an HIV-prevalent setting in Western Kenya. Xpert was performed in all patients with signs and symptoms of TB who were evaluated at the Chest Clinic. Results were rapidly available. We hypothesized that treated patients whether empirically or with confirmed TB would have a lower mortality compared to those not treated. As mortality was expected to be higher among HIV-positive patients compared to HIV-negative, we evaluated the mortality in these two groups of patients separately. We therefore assessed the mortality in the 6 months after the initial TB consultation among HIV-positive and HIV-negative patients with signs and symptoms of TB who received empirical TB treatment or TB treatment based on bacteriological confirmation and we compared it with the mortality among those who did not receive TB treatment.

## Methods

### Design and population

This analysis is part of a prospective cohort study conducted between July 2012 and June 2014 at the district level in an HIV-prevalent setting that aimed to evaluate an algorithm including the Xpert MTB/RIF assay to diagnose pulmonary TB (PTB) [23]. The study included adults (≥15 years) with signs and symptoms suggestive of PTB (i.e. presumptive PTB), able to produce sputum, who attended the outpatient TB clinic of Homa Bay County Hospital and were living in the area (maximum 20 km from the hospital). Patients were included consecutively after obtaining written informed consent. Patients who had been treated with fluoroquinolones or anti-tuberculosis drugs in the month prior to the consultation, or who were relocating out of Homa Bay in the near-term following the consultation, were excluded.

### Site

The study was conducted at the TB clinic of Homa Bay County Hospital, which is the reference health facility for a county of about 800,000 people. Kenya is a high TB burden country with an incidence of 246/100,000 in 2014; Nyanza Province, where Homa Bay is located, is the area with the highest case load reported in the country [24]. In 2013, overall HIV prevalence among people aged 15–49 years was estimated at 6.0% in the country and 25.7% in Homa Bay [25]. TB and HIV care was offered to the patients free of charge through the support of Ministry of Health and Médecins Sans Frontières.

### Procedures

On the first day, patients were screened and those fulfilling the inclusion criteria and having no exclusion criteria were included in the study. Patients included in the study were assessed clinically and requested to produce two sputum samples (one on spot and one early morning on the following day). All samples were processed on the same day of collection or the day after. The spot sample was processed for smear microscopy, Xpert MTB/RIF assay and *Mycobacterium tuberculosis complex* (MTB) culture. The early morning sample was processed for microscopy and MTB culture. Microscopy and Xpert results were received on the same day or the day after sample collection. Patients with positive microscopy or Xpert were started on treatment. Those with negative results had a chest X-ray performed at the hospital. The study clinical officer on site read and interpreted the films and made a TB treatment decision based on the clinical exam and the chest X-ray. Patients not started on TB treatment were given a broad spectrum antibiotic targeting community-acquired pneumonia and were reassessed clinically after 5 days [26]. During the re-assessment consultation, patients with partial or no clinical response had additional smear microscopy and Xpert assessment on sputum. At this point, the study clinical officer made a TB treatment decision based on the clinical exam and the additional Xpert result. All patients, whether on TB treatment or not, were requested to return at the TB clinic for clinical assessment by the clinical officer 2 and at 6 months after the first consultation. For empirically treated patients, TB treatment was not stopped after receiving a negative culture result.

Patients missing appointments and patients with positive MTB culture result who had not been started on TB treatment were traced by phone and in-person, and asked to return to the clinic. Outcome (alive/dead) at 6 months was assessed through tracing for patients who missed their 6-month consultation appointment. HIV testing with pre- and post-test counselling was proposed to all patients. Patients diagnosed with HIV infection were offered HIV care including clinical follow-up, CD4 monitoring, ART initiation, counselling and other laboratory investigations as necessary.

Following the usual procedures for TB diagnosis in Homa Bay District Hospital, the chest X-rays were interpreted by a clinical officer and not by a medical officer. However, the X-ray films were sent in batches to a radiologist for quality control purposes. The radiologist did not have information about the patient’s symptoms. A tick sheet including pictograms was used for reporting X-ray results by the treating clinical officer and the radiologist.

Sputum samples to be tested with Xpert and culture were centrifuged at 3000 rpm for 15 ± 20 min and decontaminated using 2% NALC-NaOH method. At least 0.5 ml of the sediment was resuspended in a conical tube by adding 1.5 ml of Xpert MTB/RIF sample reagent. The suspension was incubated for 15 min at room temperature before being added to a cartridge and processed. The test was repeated up to two times using a new cartridge in case of an invalid, error or no result. Samples collected for culture were processed on the same day of collection or kept in a fridge until the next day. Culture was performed using 2 methods: Thin Layer Agar (TLA) and Lowenstein-Jensen (LJ). TLA method consists of plates of 7H11 agar-based medium read by conventional microscope [27]. Sputum specimens were decontaminated as described above the re-suspended sediment was inoculated in one TLA plate and in one LJ slant. TLA was incubated at 37 °C in a 5% CO2 incubator and LJ at 37 °C in a standard incubator. Para-nitrobenzoic acid (PNB) was included in the TLA plates for simultaneous non-tuberculous mycobacteria (NTM) detection while the antigen test MPT64 was used for the identification of MTB growth in LJ. Final culture results were reported by the laboratory as positive if any of the 4 culture results was positive, negative if at least 2 results were negative and as contaminated if at least 3 results were contaminated. Negative culture patients included those positive for NTM. Inconclusive culture results comprised contaminated and missing results.

### Data collection, sample size and statistical analyses

Patient’s data was collected using hard-copy case report forms specifically designed for the study. The study collected the following information: demographics, HIV status, CD4 count for HIV infected patients, symptoms and findings of the physical exam, antibiotic prescription (if any), microscopy results, Xpert MTB/RIF results, radiological findings, MTB culture results, decision to initiate TB treatment and date, clinical situation and outcome at 6 months, date of death if deceased, and tracing outcome (if tracing done). Data were double entered at the study site using Epi-Data 3.0 software (The EpiData Association, Odense Denmark), with verification of data entry by cross checking of the 2 databases and correction of discrepancies with the case report form.

There was no sample size calculation for this analysis. All patients enrolled in the main diagnostic cohort study were included in this secondary analysis [23].

Patient characteristics were summarized using frequencies and percentages for categorical variables, and median and interquartile ranges (IQRs) for continuous variables. Mortality in the 6 months after the first consultation was the primary endpoint and TB treatment decision the main variable of interest. Patients were divided in 3 groups according to TB treatment decision: “Empirical TB treatment”, “TB treatment with confirmed TB”, “No TB treatment”.

“Empirical TB treatment” was defined as patients who received TB treatment and had no bacteriological confirmation (negative Xpert or culture result). “TB treatment with confirmed TB” was defined as patients who received TB treatment and had bacteriologically confirmed TB (positive Xpert or culture result) [28]. “No TB treatment” was defined as patients who did not receive TB treatment (none of the patients in this category had a positive Xpert or culture result).

Patients were considered severely ill when presenting at least one of the following signs: temperature higher than 39 °C, respiratory rate higher than 30 respirations/minute, heart rate higher than 120 beats minute or unable to walk without help.

Mortality in the 6 months after the first TB consultation was explored using Kaplan-Meier estimates and incidence rates stratified by HIV status and TB treatment decision (categorized as: No TB treatment, empirical treatment, treatment with confirmed TB). Comparisons of Kaplan-Meier mortality curves were done using log-rank test. Univariate and multivariate Cox proportional hazard model was performed separately for HIV-positive and HIV-negative patients to assess the association between mortality and TB treatment decision. Covariates for adjustment included: gender, age (per 1 year increase), clinical severity (severely ill or not), Body Mass Index (< 17 vs ≥17) and TB treatment history. In addition, CD4 (< 200 vs ≥200) and ART initiation (ART treatment started before and ART treatment started during the study vs not started) were included in the model for HIV-positive patients. The result of the chest X-ray interpretation by the clinical officer was included in the univariate analyses only as it could be associated with a decision of empirical treatment. Missing data were not imputed. Proportional hazards (PH) assumption was checked by testing the Schoenfeld residuals. Final multivariate models were fitted using a backward stepwise approach. Statistical significance was assessed with the likelihood ratio test at the 5% level. Data were analysed in Stata® 15.0 software (College Station, Texas, USA).

### Ethical considerations

The study protocol was approved by the KEMRI/Scientific and Ethics Review Committee in Kenya and the Comité de Protection des Personnes (CPP), Saint Germain en Laye, France.

## Results

### Characteristics of the study population

**Fig. 1:**
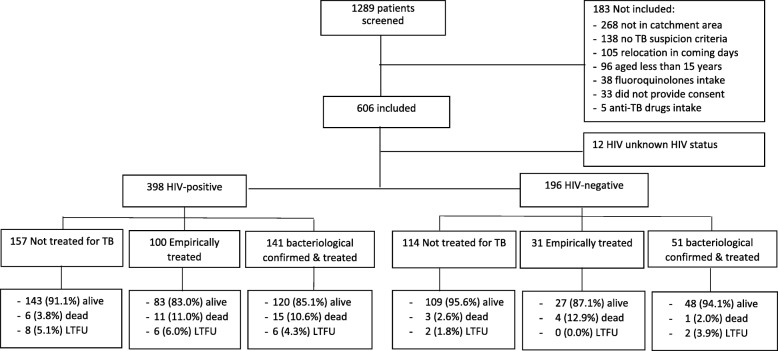
Patient flow, TB treatment decision and outcomes according to the HIV-status (LTFU: lost to follow-up)

In total, 1055 symptomatic adult patients were screened at the TB clinic and 606 (57.4%) were included in the study (Fig. 1). Of them, 344/606 (56.8%) were women and median age was 35 years [Interquartile Range (IQR): 27–47]. In total, 155/606 (25.6%) had a previous history of TB and 170/606 (28.1%) had received an antibiotic treatment in the 2 weeks prior the TB consultation. Median BMI was 19 (IQR: 17–21), 144 (23.8%) had a BMI below 17Kg/m^2^, 76 (12.5%) a BMI below 16Kg/m^2^ and 94/606 (15.5%) were severely ill.

Overall, 398/594 (67.0%) patients were HIV-positive and 196/594 (33.0%) HIV-negative. Of the HIV-positive, 319/398 (80.2%) knew their HIV status on arrival at the TB clinic. Of the 218 patients who did not know their HIV status before consultation, 206/218 (94.5%) accepted the test and 79/206 (38.4%) of them were found positive. Median CD4 count was 286 cells/μl (IQR: 137–464) and 129/376 (34.3%) had less than 200 cells/μl. This proportion was similar among those who knew their HIV status and those who did not: 32.9% vs 30.4% (*p* = 0.666). Regarding antiretroviral therapy (ART), 317/398 (79.6%) HIV-positive patients had ever initiated treatment, 184/317 (58.0%) of them before the TB clinic consultation. Chest X-ray was interpreted by the site clinician as suggestive of TB in 64/427 (15.0%) patients. In total, 178/585 (30.4%) patients had a positive culture result and 172/604 (28.5%) patients had a positive Xpert result, 2/172 (1.2%) of them were resistant to rifampicin. Culture results were inconclusive in 21/585 (3.5%) patients, including 18/585 (3.0%) culture-contaminated results and 3/585 (0.5%) missing. In 1/585 (0.2%) case NTM was found. Overall, 196/606 (32.3%) patients had a positive Xpert or culture result.

### TB treatment decision

Of the 606 patients with signs and symptoms of TB, 275 (45.4%) did not receive TB treatment (all of them were culture and/or Xpert negative). Among the 331/606 (54.6%) patients who were started on TB treatment, 135/331 (40.8%) received empirical TB treatment and 196/331 (59.2%) were bacteriologically confirmed with TB. Among the 398 HIV-positive patients, 157 (39.5%) did not received treatment, 100 (25.1%) received empirical treatment and 141 (35.4%) received treatment based on bacteriologically confirmed TB. Among the 196 HIV-negative patients, 114 (58.2%) did not received treatment, 31 (15.8%) received empirical treatment and 51 (26.0%) received treatment based on bacteriologically confirmed TB. Among the patients treated for TB, the proportion of patients who received empirical TB treatment was not statistically significant different among HIV-positive and HIV-negative: 100/241 (41.5%) vs 31/82 (37.8%), *p* = 0.557. However, excluding the patients with bacteriologically-confirmed TB, the proportion of empiric treatment was higher among HIV-positive patients than among HIV-negative: 100/257 (38.9%) vs 31/145 (21.4%), *p* < 0.001. In total, 292/331 (88.2%) patients started treatment at the first consultation, 30/331 (9.1%) at the second consultation after clinical re-assessment and repeated smear microscopy and Xpert, and 9/331 (2.7%) after tracing due to a positive culture result. The characteristics of the patients according to the TB treatment decision are presented in Table 1.

**Table 1:** Characteristics of the patients according to the treatment decision for tuberculosis and the HIV status

	HIV-positive (*N* = 398)			HIV-negative (*N* = 196)		
	No treatment (*n* = 157)	Empirical treatment (*n* = 100)	Treatment based on confirmed TB (*n* = 141)	No treatment (*n* = 114)	Empirical TB treatment (*n* = 31)	Treatment based on confirmed TB (*n* = 51)
Age, median years [IQR]	36 [30–45]	34 [27–42]	32 [26–38]	45.5 [28–59]	58 [34–70]	28 [22–37]
Women	71.3	51.0	48.2	64.9	38.7	43.1
History of tuberculosis	40.1	31.0	12.8	30.7	9.7	7.8
Antibiotic prior consultation	27.4	34.0	22.9	25.4	35.5	31.4
BMI^a^, median [IQR]	19.1 [17.8–21.0]	18.1 [16.2–19.8]	17.5 [16.0–19.1]	20.4 [18.4–23.1]	19.3 [17.0–20.8]	18.8 [17.4–19.8]
Moderate/Severe thinness (BMI < 17 Kg/m^2^)	14.0	33.0	44.0	7.0	29.0	11.8
Severely ill	3.9	25.3	31.4	1.8	16.7	19.6
Chest X-ray suggestive of TB^b^	0.0	41.1	30.8	0.0	45.2	40.0

^a^*BMI* body mass index

^b^Chest X-ray performed: 273 HIV-positive, 147 HIV-negative

### Mortality in the six months after the first consultation, and association with TB treatment

Of the 606 patients included, 42 (6.9%) died in the 6 months following the first consultation: 32/398 (8.0%) among HIV-positive and 8/196 (4.1%) among HIV-negative patients (*p* = 0.070). A total of 27 (4.5%) were lost to follow-up. Mortality was 1.3 (95%CI: 0.9–1.7) deaths/100 patient-months: 1.6/100 patient-months (95%CI: 1.1–2.2) among HIV-positive patients and 0.8/100 patient-months (95%CI: 0.4–1.5) among HIV-negative. Median time to death was 2.0 months [IQR 1.2–4.7] in HIV-positive patients and 3.3 months [IQR 2.7–4.6] in HIV-negative. Among patients who received empiric TB treatment, median time to death was 1.4 months [IQR 0.8–3.7] in HIV-positive patients and 3.3 months [IQR 3.2–4.5] in HIV-negative.

**Fig. 2:**
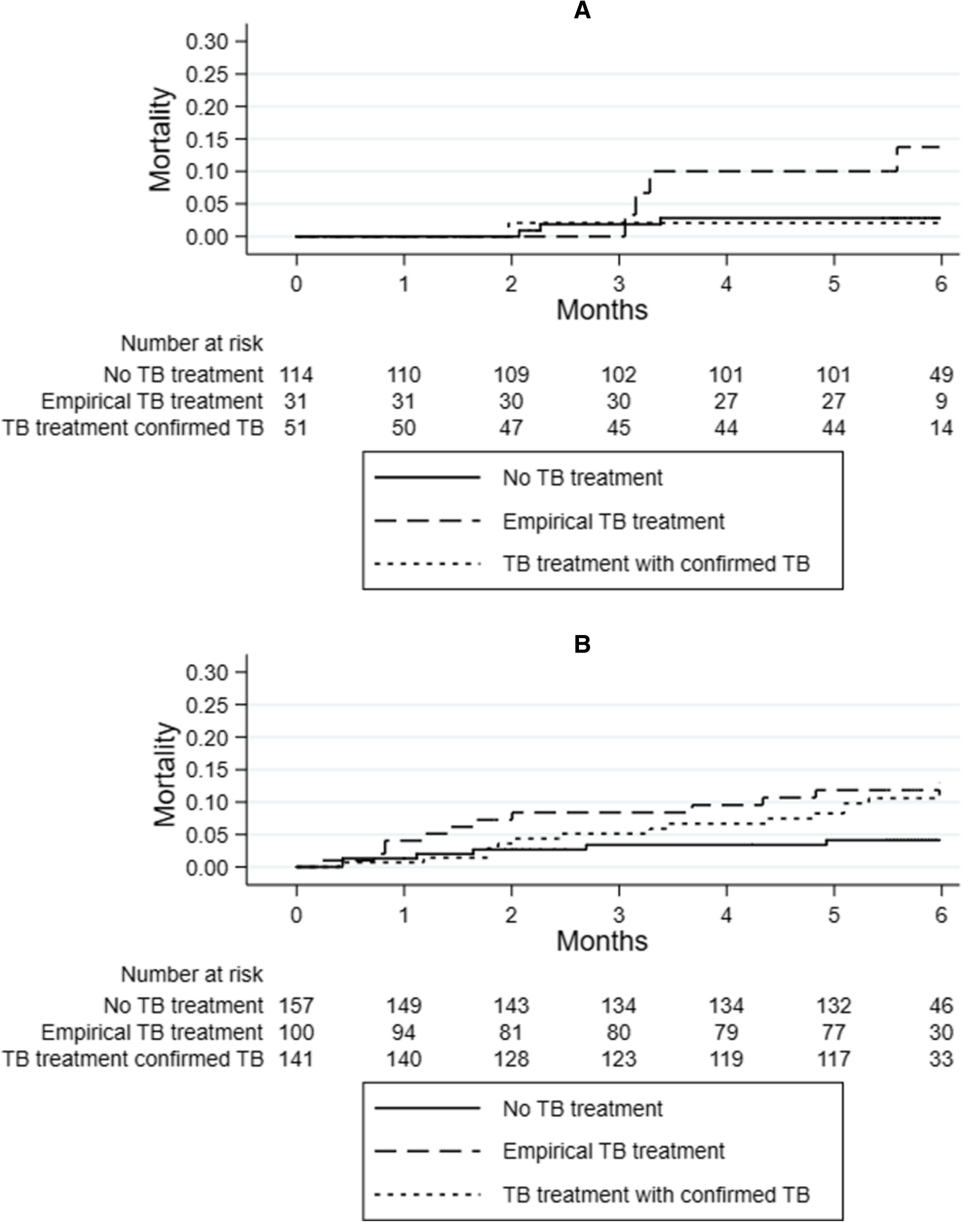
Kaplain-Meier estimates of the mortality in the six months after the first consultation in HIV-negative (**a**) and HIV-positive patients (**b**) with signs and symptoms of TB according to the TB treatment decision: not started on TB treatment, empirical TB treatment, TB treatment based on bacteriological confirmation

Kaplan-Meier estimates of mortality according to treatment start and HIV-status are displayed in Fig. 2. Among HIV-positive patients, the mortality in the 6 months after the first consultation was higher in patients who received empirical treatment and in patients treated based on bacteriological confirmation compared to those who did not receive TB treatment: 11.8% (95%CI 6.7–20.4), 13.1% (95%CI 7.6–22.1) and 4.1% (95% CI 1.9–9.0), respectively (*p* = 0.049). Among HIV-negative patients, the mortality in the 6 months after the first consultation was higher in patients who received empirical treatment compared to those with confirmed TB and those who did not received TB treatment: 13.8% (95%CI 5.4–32.7), 2.1% (95%CI 0.3–13.9) and 2.8% (95% CI 0.9–8.5), respectively (*p* = 0.033).

Results of the univariate and multivariate Cox model are shown in Table 2. After adjustment, severe illness, BMI below 17 Kg/m^2^ and CD4 count below 200 cells/μL were independently associated with a higher mortality among HIV-positive patients. In the multivariate model, TB treatment, whether empirical or based on bacteriological confirmation, was not associated with increased mortality among HIV-positive patients (aHR: 2.51, 95%CI: 0.79–7.90 and aHR: 1.25, 95%CI: 0.37–4.21 respectively). However, HIV-negative patients who received empirical treatment had a higher risk of mortality (aHR: 4.85, 95%CI: 1.08–21.67) compared to those not started on TB treatment. HIV-negative patients who received TB treatment based on bacteriological confirmation did not have an increased risk of mortality (aHR: 0.77, 95%CI: 0.08–7.41).

**Table 2:** Univariate and multivariate Cox regression analysis of the association between treatment decision for tuberculosis and mortality in the six months after the first consultation in HIV-positive and HIV-negative patients with signs and symptoms of TB

			HIV-positive (N = 398)							HIV-negative (N = 196)				
		Unadjusted				Adjusted				Unadjusted		Adjusted		
	Deaths n/N (%)	HR	95% CI	p	aHR	95% CI	p	Deaths n/N (%)	HR	95% CI	p	aHR	95% CI	p
Age (per additional year)		0.98	0.95–1.02	0.303	–	–	–		1.03	0.99–1.07	0.142	–	–	–
Sex														
Men	15/152 (9.0)	Ref						4/88 (4.5)	Ref					
Women	17/231 (7.4)	0.81	0.40–1.62	0.550	–	–	–	4/108 (3.7)	0.79	0.20–3.15	0.736	–	–	–
BMI (Kg/m^**2**^)														
> =17	11/281 (3.9)	Ref			Ref			6/173 (3.5)	Ref					
< 17	21/117 (18.0)	4.91	2.36–10-18	< 0.001	3.55	1.51–8.35	0.004	2/23 (8.7)	2.41	0.49–11.93	0.282	–	–	–
Severely ill														
No	15/320 (4.7)	Ref			Ref			7/178 (3.9)	Ref			–		
Yes	17/75 (22.7)	5.32	2.66–10.66	< 0.001	2.67	1.17–6.12	0.020	1/17 (5.9)	1.49	0.18–12.10	0.710	–	–	–
TB history														
No	19/286 (6.6)	Ref						7/154 (4.5)	Ref					
Yes	13/112 (11.6)	1.86	0.92–3.76	0.086	–	–	–	1/42 (2.4)	0.51	0.06–4.17	0.532	–	–	–
CD4 count (cells/mL)														
> =200	9/247 (3.6)	Ref			Ref				–	–	–	–	–	–
< 200	20/129 (15.5)	4.54	2.06–9.97	< 0.001	3.21	1.42–7.28	0.005		–	–	–	–	–	–
ART														
Not initiated	6/58 (10.3)	Ref			Ref				–	–	–	–	–	–
Initiated before study	17/184 (9.2)	0.82	0.32–2.09	0.680	0.74	0.27–2.07	0.573		–	–	–	–	–	–
Initiated during study	6/132 (4.5)	0.37	0.12–1.14	0.084	0.19	0.05–0.65	0.008		–	–	–	–	–	–
Chest X-ray														
Normal	3/62 (4.8)	Ref												
Possible TB	12/164 (7.3)	1.55	0.43–5.50	0.496	–	–	–		–	–	–	–	–	–
Suggestive TB	3/47 (6.4)	1.31	0.26–6.48	0.742	–	–	–		–	–	–	–	–	–
TB treatment														
No TB treatment^a^	6/157 (3.8)	Ref			Ref			3/114 (2.6)	Ref			Ref		
Empirical treatment	11/100 (11.0)	3.04	1.12–8.21	0.029	2.51	0.79–7.90	0.115	4/31 (12.9)	4.85	1.08–21.67	0.039	4.85	1.08–21.67	0.039
Treatment based on confirmed TB	15/141 (10.6)	2.76	1.07–7.12	0.036	1.25	0.37–4.21	0.718	1/51 (2.0)	0.77	0.08–7.41	0.822	0.77	0.08–7.41	0.822

^a^Patients who did not receive TB treatment were all culture and/or Xpert negative

The demographic characteristics, clinic-radiological findings and management of the 8 HIV-negative patients who died are presented in Table 3. Three of four deceased patients started empirically on TB treatment were aged 65 years or more. All were started on TB treatment at the first consultation and this decision was based on the chest X-ray interpreted by the treating clinical officer. However, the radiologist who did a second reading for quality control only interpreted one X-ray as possible TB. Two patients had a high blood pressure on clinical examination, the radiologist described cardiomegaly on their chest X-ray and concluded that there were no radiological signs of TB. One of them also had oedema in the lower limbs. This clinical and radiological findings may indicate a cardiovascular disease in these two patients.

**Table 3:** Demographic characteristics, clinical-radiological findings and management of the 8 HIV negative deceased patients

	Age range (years)	Sex	BMI (Kg/m^2^)	Temperature (°C)	Respiratory rate (/min)	Pulse (/min)	Blood Pressure (mmHg)	Oedema in lower limbs	Xpert result	TB treatment	Reason to start TB treatment	Chest X-ray interpretation and conclusion by site clinician	Chest X-ray interpretation and conclusion by radiologist (quality control)	Symptoms’ assessment at 2 months	Management at 2 months	Days to death
Patient 1	31–35	M	21.5	36.7	24	81	228/149	Yes	Neg	Treated empirically at first consultation	X-ray	Infiltrates, adenopathies; Possible TB	Cardiomegalia, Empyema, Brochiectasis, Not TB	No resolution	Stop TB treatment	96
Patient 2	64–65	M	17.4	36.2	20	71	130/67	No	Neg	Treated empirically at first consultation	X-ray	Infiltrates; Possible TB	Infiltrates, Possible TB	No resolution	Continue TB treatment	100
Patient 3	71–75	M	17.8	36.5	28	100	140/70	No	Neg	Treated empirically at first consultation	X-ray	Infiltrates, adenophathies, consolidation, cavities; Definitively TB	Consolidation, Fibrosis, Not TB	Complete resolution	Continue TB treatment	170
Patient 4	71–75	F	22.5	36.0	26	76	170/90	No	Neg	Treated empirically at first consultation	X-ray	Infiltrates, adenopathies, consolidation, pleural effusion; Definitively TB	Pleural effusion, Cardiomegalia, Not TB	Partial resolution	Continue TB treatment	93
Patient 5	21–25	F	17.8	36.9	29	95	96/63	No	Pos	Treated after bacteriological confirmation at first consultation	Xpert	Not done	Not done	Partial resolution	Continue TB treatment	60
Patient 6	35–40	F	13.8	36.7	34	103	81/38	No	Neg	Not treated	–	Missing data	No abnormal findings; Not TB	Not seen; Hospitalized	–	69
Patient 7	41–45	F	21.0	36.9	28	107	140/88	No	Neg	Not treated	–	Infiltrates, adenopathies, cardiomegalia; Possible TB	Cardiomegalia; Not TB	Complete resolution	No specific treatment	182
Patient 8	75–80	M	16.6	36.6	28	90	120/68	Yes	Neg	Not treated	–	Infiltrates, adenopathies, cavities; Possible TB	Granuloma, pleural thickening; Not TB	Not seen; Bed ridden at home	–	103

## Discussion

HIV-positive patients with signs and symptoms of TB who received TB treatment, whether empirically or based on bacteriological confirmation, did not have a different risk of mortality in the 6 months after the first consultation, compared to those not treated after adjustment for the clinical severity of the patients’ condition and their immunological status. However, the risk of mortality was increased among HIV-negative patients treated empirically compared to those not treated, while treated patients who had bacteriologically confirmed TB did not have a different risk of mortality.

An important proportion of the patients who received TB treatment were treated empirically despite having Xpert results available on the day of consultation or the day after. There were more patients who were severely ill or had moderate/severe thinness among patients empirically treated for TB compared to those not treated. This suggests that severity of clinical condition may influence clinicians’ decision to treat empirically. In Brazil, HIV-positive patients with cough who were smear and culture negative and received TB treatment had a higher mortality than those who did not [29]. The authors interpreted that even after adjustment, severe HIV disease remained a confounder of the association between empirical TB treatment and mortality. In our study, among HIV-positive patients, the clinical status of those treated for TB whether empirically or based on bacteriological confirmation may partially explain the increased crude mortality since after adjustment for clinical signs of severe illness, BMI, CD4 and ART, TB treatment was not associated with a higher mortality. Similarly, in a study conducted in Uganda, smear-negative HIV-positive patients started on empirical TB treatment had a higher risk of mortality at 1 year in a simple mortality analyses. However after adjustment for CD4 count and ART, no association between empirical treatment and death was found [21]. In contrast with the results previously mentioned, another study in Uganda reported an adjusted relative risk reduction of 44% in the 8-week mortality of HIV-positive patients with clinical signs of severe illness who received empirical treatment [18]. However, this latest study was conducted before Xpert was available which is an important distinction compared to the studies previously mentioned including our study, in terms of the population receiving empirical treatment. In addition, the mortality was assessed at 8-weeks which may be not comparable with the mortality at 6 months or 1 year.

The high risk of undiagnosed TB and associated mortality has supported the hypothesis that routine systematic TB treatment could be useful in a subset of asymptomatic or paucisymptomatic HIV-patients with low CD4 [30]. The results of REMEMBER [19] and STATIS trials [31], two clinical trials that assessed this question, do not seem to support this hypothesis. Systematic empiric TB therapy, after exclusion of patients with confirmed or suspected TB, did not reduce mortality at 24 weeks in asymptomatic outpatient adults with advanced HIV disease initiating ART compared to receiving systematic TB screening and IPT in REMEMBER or intensified TB screening in STATIS. Moreover, in REMEMBER there was a suggestion of possible harm in the empiric arm with respect to incident TB and HIV disease progression. Although, the trial population of asymptomatic HIV-advanced outpatients was different from our study population, the results highlighted that clinicians, by making a decision to give TB treatment, may not further investigate other concomitant pathologies that could potentially lead to death. Other authors have also considered that, in addition to possible treatment adverse events, missing other non-TB diagnoses could be one of the potential adverse consequences of the empirical treatment [30]. This does seem to be the case in our study among HIV-positive patients but could be particularly important among HIV-negative patients. In addition, some of the deceased HIV-negative patients who had received empirical treatment presented clinical and radiological signs of cardiovascular disease. Our hypothesis is that misdiagnosis in some cases may have occurred in HIV-negative patients. In this population, other severe conditions not captured in the analyses may have been present and contributing to the higher mortality.

This study has some limitations. The number of deaths in the HIV-negative group was low. Although the association between empirical treatment and mortality was statistically significant, the hazard rate estimation should be carefully interpreted as its confidence interval is large. In addition, there may be other potential confounding factors of the relationship between empirical treatment and mortality than those included in the analyses of HIV-positive and HIV-negative patients. This evaluation was conducted in a unique site which limits the generalization of the findings. Although in a small proportion, some of the patients were lost to follow-up. In addition, the causes of death were not investigated.

## Conclusion

TB treatment decision on patients with signs and symptoms of TB and negative or no Xpert results is challenging in resource-limited settings. Our findings suggest that in a context with access to Xpert, clinicians should continue using empirical TB treatment in HIV-positive patients with signs and symptoms of TB and negative Xpert results since there is a proportion of TB cases that cannot be confirmed by Xpert. However, differential diagnoses other than TB should be actively sought before initiating empirical TB treatment, particularly in HIV-negative patients with Xpert negative results. In addition, clinicians should be alert to other possible diagnoses during the follow-up of patients on TB treatment when there is poor response, particularly for those who do not have microbiological confirmation of TB.

## Abbreviations

ART: Antiretroviral therapy
LJ: Lowenstein-Jensen
MTB: *Mycobacterium tuberculosis complex*
NTM: Non-tuberculous mycobacteria
PNB: Para-nitrobenzoic acid
PTB: pulmonary tuberculosis
TB: tuberculosis
TLA: Thin Layer Agar
Xpert: GeneXpert MTB/RIF
